# *Saussurea lappa* extract suppresses TPA-induced cell invasion via inhibition of NF-κB-dependent MMP-9 expression in MCF-7 breast cancer cells

**DOI:** 10.1186/1472-6882-14-170

**Published:** 2014-05-25

**Authors:** Ha-Rim Kim, Jeong-Mi Kim, Mi-Seong Kim, Jin-Ki Hwang, Yeon-Ju Park, Sei-Hoon Yang, Hye-Jung Kim, Do-Gon Ryu, Dong-Sung Lee, Hyuncheol Oh, Youn-Chul Kim, Yun-Jin Rhee, Byung-Soon Moon, Jong-Min Yun, Kang-Beom Kwon, Young-Rae Lee

**Affiliations:** 1 Center for Metabolic Function Regulation, Wonkwang University School of Medicine, Iksan 570-749, South Korea; 2 Department of Internal Medicine, Wonkwang University School of Medicine, Iksan 570-749, South Korea; 3 Department of Family Medicine, The Catholic University of Korea, Incheon St. Mary's Hospital, Incheon 403-720, South Korea; 4 Department of Korean Physiology, Wonkwang University School of Korean Medicine, Iksan 570-749, South Korea; 5 Hanbang Body-Fluid Research Center, Wonkwang University, #460 Iksan-daero, Iksan, Jeonbuk 570-749, South Korea; 6 Standardized Material Bank for New Botanical Drugs, College of Pharmacy, Wonkwang University, #460 Iksan-daero, Iksan, Jeonbuk 570-749, South Korea; 7 Institute of Pharmaceutical Research and Development, College of Pharmacy, Wonkwang University, #460 Iksan-daero, Iksan, Jeonbuk 570-749, South Korea; 8 Department of Oral Biochemistry, and Institute of Biomaterials, Implant, School of Dentistry, Wonkwang University, Iksan 570-749, South Korea; 9 Department of Internal Medicine, College of Korean Medicine, Wonkwang University, #460 Iksan-daero, Iksan City, Jeonbuk 570-749, South Korea; 10 BK21plus program & Department of Smart Life-Care Convergence, Wonkwang University, Graduate School, #460 Iksan-daero, Iksan City, Jeonbuk 570-749, South Korea

**Keywords:** *Saussurea lappa*, MMP-9, Invasion, NF-κB, MCF-7

## Abstract

**Background:**

*Saussurea lappa* (SL) has been used as a traditional herbal medicine to treat abdominal pain and tenesmus, and has been suggested to possess various biological activities, including anti-tumor, anti-ulcer, anti-inflammatory, anti-viral, and cardiotonic activities. The effect of SL on breast cancer metastasis, however, is unknown. Cell migration and invasion are crucial in neoplastic metastasis. Matrix metalloproteinase-9 (MMP-9), which degrades the extracellular matrix, is a major component in cancer cell invasion.

**Methods:**

Cell viability was examined by MTT assay, whereas cell motility was measured by invasion assay. Western blot, Real-time PCR, and Zymography assays were used to investigate the inhibitory effects of ESL on matrix metalloproteinase-9 (MMP-9) expression level in MCF-7 cells. EMSA confirmed the inhibitory effects of ESL on DNA binding of NF- κB in MCF-7 cells.

**Results:**

Cells threated with various concentrations of Saussurea lappa (ESL) for 24 h. Concentrations of 2 or 4 μM did not lead to a significant change in cell viability or morphology. Therefore, subsequent experiments utilized the optimal non-toxic concentration (2 or 4 μM) of ESL. In this study, we investigated the inhibitory effect of ethanol extract of ESL on MMP-9 expression and cell invasion in 12-*O*-tetradecanoylphorbol-13-acetate (TPA)-induced MCF-7 cells. ESL inhibited the TPA-induced transcriptional activation of nuclear factor-kappa B (NF-κB). However, this result obtained that ESL did not block the TPA-induced phosphorylation of the kinases: p38, ERK, and JNK. Therefore, ELS-mediated inhibition of TPA-induced MMP-9 expression and cell invasion involves the suppression of NF-kB pathway in MCF-7 cells.

**Conclusions:**

These results indicate that ELS-mediated inhibition of TPA-induced MMP-9 expression and cell invasion involves the suppression of NF-kB pathway in MCF-7 cells. Thus, ESL has potential for controlling breast cancer invasiveness in vitro.

## Background

Breast cancer is one of the leading causes of malignancy-related death in women [1]. Most breast cancer deaths are caused by distant metastasis from the primary tumor site. Failure in its treatment mainly arises from cancer proliferation, invasion, and metastasis, which ultimately lead to the death of patients. Penetration of the extracellular basement membrane by cells is the premise of cancer cell metastasis, where a variety of proteases play essential roles. Invasion and metastasis are the fundamental properties and major causes of morbidity and mortality in breast cancer patients. Molecular mechanisms of cancer cell invasion and metastasis involve a complex series of events. One such early event involves proteolytic degradation of the components of the extracellular matrix (ECM) [2], which provides biochemical and mechanical barriers to cell movement in cancer cells [3]. The ECM consists of type IV collagen, laminin, heparan sulfate proteoglycan, nidogen, and fibronectin [4]. ECM degradation requires extracellular proteinases, of which the matrix metalloproteinases (MMPs) have been shown to play a critical role in breast cancer.

MMPs are a major group of enzymes that regulate cellular matrix composition, and are zinc- and calcium-dependent endopeptidases. Based on their substrates, MMPs are classified into four subclasses including collagenases, gelatinases, and stromelysins. MMP-9 in particular is considered to be one of the critical MMPs involved in cancer invasion and has been found to be directly associated with the invasion, metastasis, and poor prognosis of breast cancer [5,6]. Therefore, inhibiting MMP-9 expression and/or its upstream regulatory pathways might be critical in treating malignant tumors, including breast carcinoma. A variety of stimuli, including growth factors (e.g., fibroblast growth factor-2, epidermal growth factor, and hepatocyte growth factor), cytokines (e.g., tumor necrosis factor-alpha), oncogenes (e.g., Ras), and 12-*O-*tetradecanoylphorbol-13-acetate (TPA) are known to induce MMP-9 expression [7-11]. Among these stimuli, TPA is a well-known selective activator of protein kinase C (PKC) [8] and can stimulate MMP-9 synthesis and secretion in breast cancer cell invasion [12,13]. Cytokine and TPA treatments induce MMP-9 expression via activation of transcription factors such as nuclear factor-kappa B (NF-κB) and activator protein-1 (AP-1) [14-16], both of which have binding sites on the MMP-9 gene promoter [17]. The NF-κB and AP-1 elements are centrally involved in MMP-9 gene induction by TPA [14,15]. The mitogen-activated protein kinase (MAPK) signaling pathway is important for AP-1 activation, and NF-κB activation requires I-κB kinase, extracellular signal-regulated kinase (ERK), c-Jun N-terminal kinase (JNK), or p38 MAPK, depending on the cell type [11,18,19].

*Saussurea lappa* (SL) is indigenous to India and Pakistan and has been cultivated in Southwest China, where it is utilized as a medicine. The dried roots of *S. lappa* have been traditionally used to alleviate pain from abdominal distention and tenesmus, anorexia-associated indigestion, dysentery, nausea, and vomiting [20]. Previous in vitro cell culture studies have shown that SL has anti-ulcer [21], anti-inflammatory [22], anti-viral [23], and anti-tumor properties [24,25]. In addition, SL inhibits the growth of several types of cancer cells [20,26,27]. However, the mechanism by which SL mediates anti-invasiveness is not well understood. A recent study showed that SL inhibits the cytokine-induced activation of NF-κB [28], a transcription factor that is important in the regulation of MMP-9. Accordingly, it has been hypothesized that SL may have anti-metastasis properties based on findings of the inhibition of cell invasion by SL. In this study, we addressed this hypothesis by assessing the potential effects of SL on TPA-induced cell invasion and MMP-9 expression in MCF-7 human breast cancer cells with related molecular mechanisms. Our findings demonstrate that ethanol extract of SL (ESL) suppresses TPA-induced MMP-9 expression by blocking the NF-κB signaling pathways, and that the suppression of MMP-9 expression correlates with inhibited cell invasion.

## Methods

### Cells and materials

MCF-7 cells were obtained from the American Type Culture Collection (Manassas, VA, USA). Cells were cultured in Dulbecco’s modified Eagle medium (DMEM) supplemented with 10% fetal bovine serum (FBS) and 1% antibiotics at 37°C in a 5% CO_2_ incubator. TPA, 3-(4,5-dimethyl-thiazol-2-yl)-2,5-diphenyltetrazolium bromide (MTT), and anti-β-actin antibody were obtained from Sigma-Aldrich (St. Louis, MO, USA). Antibodies against p38, phosphorylated p38 (p-p38), JNK, p-JNK, ERK, p-ERK, phosphorylated c-Jun (p-c-Jun), phosphorylated I-kappa-B-alpha (p-IκBα), and phosphorylated I-kappa B kinase-alpha (p-IKKα) were purchased from Cell Signaling Technology (Beverly, MA, USA). Antibodies against MMP-9, p50, p65, IκBα, IKKα, IKKβ, PKCα, PKCδ, proliferating cell nuclear antigen (PCNA), and horseradish peroxidase (HRP)-conjugated IgG were purchased from Santa Cruz Biotechnology (Santa Cruz, CA, USA). Alpha ^32^phosphorous-labelled deoxycytidine triphosphate ([α-^32^P]dCTP) was obtained from Amersham (Buckinghamshire, UK). DMEM containing a high concentration of glucose, FBS, and phosphate-buffered saline (PBS), was obtained from Gibco-BRL (Gaithersburg, ME, USA).

### Plant material and preparation of NNMBS19

The dried root of *Saussurea lappa* (Compositae) were purchased from the University Oriental Herbal Drugstore, Iksan, Korea, in August 2010, and a voucher specimen was deposited at the Herbarium of the College of Pharmacy at Wonkwang University, Iksan, Korea. The dried root of *S. lappa* (50 g) were extracted twice with hot 70% ethanol (1 L) for 2 h at room temperature, and filtered with filter paper. The filtrate was evaporated in *vacuo* to produce a 70% ethanol extract (10.58 g, 21.2 w/w%). The 70% ethanol extract was suspended in distilled water (100 mL), followed by filtration. The residue derived from the filtration was dissolved in hot ethanol and filtered again. The filtrate was then evaporated in *vacuo* to obtain a standardized fraction of *S. lappa* (NNMBS198, 1000.3 mg, 2.01 w/w%). NNMBS198 was deposited at the Standardized Material Bank for New Botanical Drugs, College of Pharmacy at Wonkwang University.

### Determination of cell viability

The effect of ESL on MCF-7 cell viability was determined using an established MTT assay. In brief, 3 × l0^4^ cells were seeded in wells and incubated at 37°C for 24 h to allow attachment. The attached cells were untreated or treated with 1, 2, 5, 10, or 30 μg/mL ESL for 24 h at 37°C. The cells were washed with PBS prior to adding MTT (0.5 mg/mL in PBS) and incubated at 37°C for 30 min. Formazan crystals were dissolved with dimethyl sulfoxide (100 μL/well) and detected at 570 nm using a Model 3550 Microplate Reader (Bio-Rad; Richmond, CA, USA).

### Western blot analysis

MCF-7 cells (5 × 10^5^) were pre-treated with ESL (2 or 4 μg/mL) for 1 h and then incubated with TPA for 24 h at 37°C. Cells were lysed with ice-cold M-PER® Mammalian Protein Extraction Reagent (Pierce Biotechnology; Rockford, IL, USA), and protein concentration was determined using the Bradford method. Samples (20 μg) were separated by sodium dodecyl sulfate-polyacrylamide gel electrophoresis with 10% acrylamide and transferred to Hybond™ polyvinylidene fluoride membranes (GE Healthcare Life Sciences; Buckinghamshire, UK) using a western blot apparatus. Each membrane was blocked for 2 h with 2% bovine serum albumin or 5% skim milk and then incubated overnight at 4°C with 1 μg/mL of a 1:2000 dilution of primary antibody. HRP-conjugated IgG (1:2000 dilution) was used as the secondary antibody. Protein expression levels were determined by signal analysis using an image analyzer (Fuji-Film; Tokyo, Japan).

### Gelatin zymography assay

Conditioned media was collected after 24 h stimulation, mixed with non-reducing sample buffer, and electrophoresed on a polyacrylamide gel containing 0.1% (w/v) gelatin. The gel was washed at room temperature for 30 min with 2.5% (v/v) Triton X-100 solution, and subsequently incubated at 37°C for 16 h in 5 mM CaCl_2_, 0.02% (v/v) Brij, and 50 mM Tris–HCl (pH 7.5). The gel was stained for 30 min with 0.25% (w/v) Coomassie Brilliant Blue in 40% (v/v) methanol/7% (v/v) acetic acid and photographed on an image analyzer (Fuji-Film). Proteolysis was visualized as a white zone in a dark blue field. Densitometric analysis was performed using Multi Gauge Image Analysis software (Fuji-Film).

### Quantitative real-time polymerase chain reaction (qRT-PCR)

Total RNA was extracted from cells using a FastPure™ RNA Kit (TaKaRa; Shiga, Japan). RNA concentration and purity were determined by absorbance at 260/280 nm. cDNA was synthesized from 1 μg total RNA using a PrimeScript™ RT reagent Kit (TaKaRa). MMP-9 and glyceraldehyde 3-phosphate dehydrogenase (GAPDH) mRNA expression levels were determined by real-time PCR using the ABI PRISM 7900 sequence detection system and SYBR® Green (Applied Biosystems; Foster City, CA, USA). The primers were the following: MMP-9 (NM 004994) sense: 5′-CCTGGAGACCTGAGAACCAATCT-3′, antisense: 5′-CCACCCGAGTGTAACCATAGC-3′; and GAPDH (NM 002046) sense: 5′-ATGGAAATCCCATCACCATCTT-3′, antisense: 5′-CGCCCCACTTGATTTTGG-3′. To control for variation in mRNA concentration, all results were normalized to the GAPDH housekeeping gene. Relative quantification was performed using the comparative ∆∆C_t_ method according to the manufacturer’s instructions.

### Preparation of nuclear extract

MCF-7 cells (2 × 10^6^) were treated with ESL in the presence or absence of TPA for 4 h. Cells were immediately washed twice, scraped into 1.5 mL of ice-cold PBS (pH 7.5), and pelleted at 1500 × *g* for 3 min. Cytoplasmic and nuclear extracts were prepared from cells using the NE-PER® Nuclear and Cytoplasmic Extraction Reagents (Pierce Biotechnology; Rockford, IL, USA).

### Electrophoretic mobility shift assay (EMSA)

Activation of NF-κB and AP-1 was assessed by gel mobility shift assays using nuclear extracts. Oligonucleotides containing the κ-chain (κB, 5′-CCGGTTAACAGAGGGGGCTTTCCGAG-3′) or AP-1 (5′-CGCTTG ATGAGTCAGCCGGAA-3′) binding site were synthesized and used as probes for gel retardation assays. The two complementary strands were annealed and labeled with [α-^32^P]dCTP. Labeled oligonucleotides (10,000 cpm), 10 μg of nuclear extracts, and binding buffer (10 mM Tris–HCl, pH 7.6, 500 mM KCl, 10 mM EDTA, 50% [v/v] glycerol, 100 ng poly[deoxyinosinic-deoxycytidylic] acid [poly(dI · dC)], 1 mM dithiothreitol) were then incubated for 30 min at room temperature in a final volume of 20 μL. The reaction mixtures were analyzed by electrophoresis on 4% polyacrylamide gels in 0.5× Tris-borate buffer. The gels were dried and examined by autoradiography. Specific binding was controlled by competition with a 50-fold excess of cold κB oligonucleotide.

### Invasion assay

The invasion assay was carried out in 24-well chambers (8-μm pore size) coated with 20 μL Matrigel™ diluted in DMEM. The Matrigel™ coating was re-hydrated in 0.5 mL DMEM for 30 min immediately before the experiments. Cells (2 × 10^5^) were seeded in the upper chambers of wells, and chemoattractant was placed in the bottom chambers. Conditioned medium (0.5 mL) was added to the lower compartment of the invasion chamber. The chambers were incubated for 24 h. Following incubation, cells on the upper side of the chamber were removed using cotton swabs, and cells that had migrated were fixed and stained with Toluidine blue solution. Invading cells were counted in five randomly selected areas of the membrane using a light microscope. Analyzed data represent the means ± standard error (SE) from three individual experiments each performed in triplicate.

### Statistical analysis

Statistical data analysis was performed using ANOVA and Duncan’s test. Differences with p < 0.05 were considered statistically significant.

## Results

### Effect of ESL on the viability of MCF-7 cells and MDA-MB-231 cells

**Figure 1:**
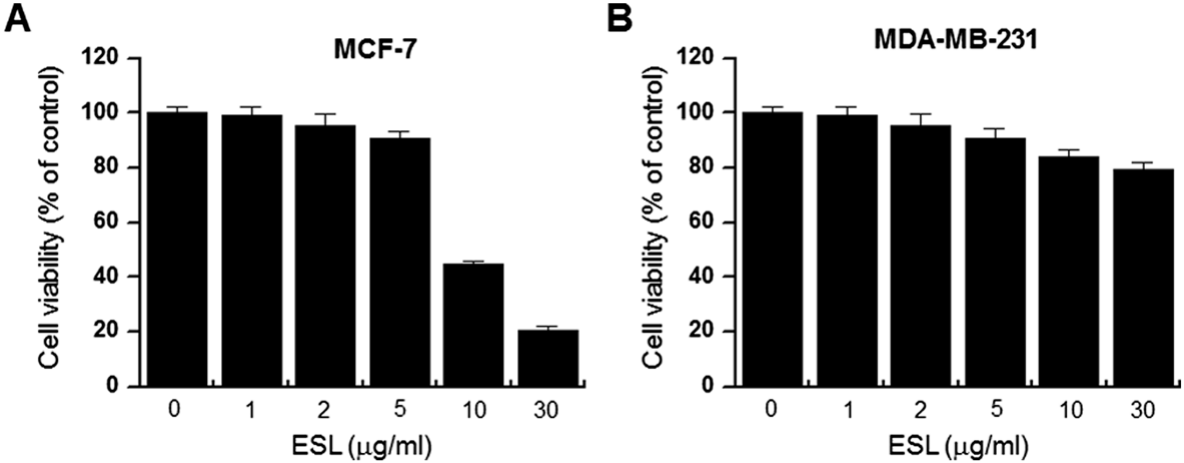
**The effect of ethanol extract of Saussurea lappa (ESL) on the viability of MCF-7 cells and MDA-MB-231 cells.** To test the cytotoxicity of ESL, MCF-7 **(A)** and MDA-MB-231 **(B)** cells were cultured in 96-well plates until 70% confluence and treated with various concentrations (0, 1, 2, 5, 10, and 30 μg/mL) of ESL for 24 h. An MTT assay was used to assess the viability of the cells, where the optical density value of an untreated control was regarded as 100% viability. Data represent the mean ± SE of three independent experiments.

To verify the effect of ESL on MCF-7 cell viability, we treated cells with the extract at the concentrations indicated for 24 h. The toxicity of ESL to MCF-7 cells was assessed using the MTT assay. MCF-7 cell viability was not affected by ESL at the concentrations tested (Figure 1A), and thus, for all subsequent experiments, ESL was used at concentrations of 2 or 4 μM. Additionally, we investigated the effect of ESL on MDAMB-231 cell viability. MDA-MB-231 cell viability was not affected by ESL at the concentrations tested (Figure 1B).

### Effect of ESL on TPA-induced MMP-9 expression in MCF-7 cells

**Figure 2:**
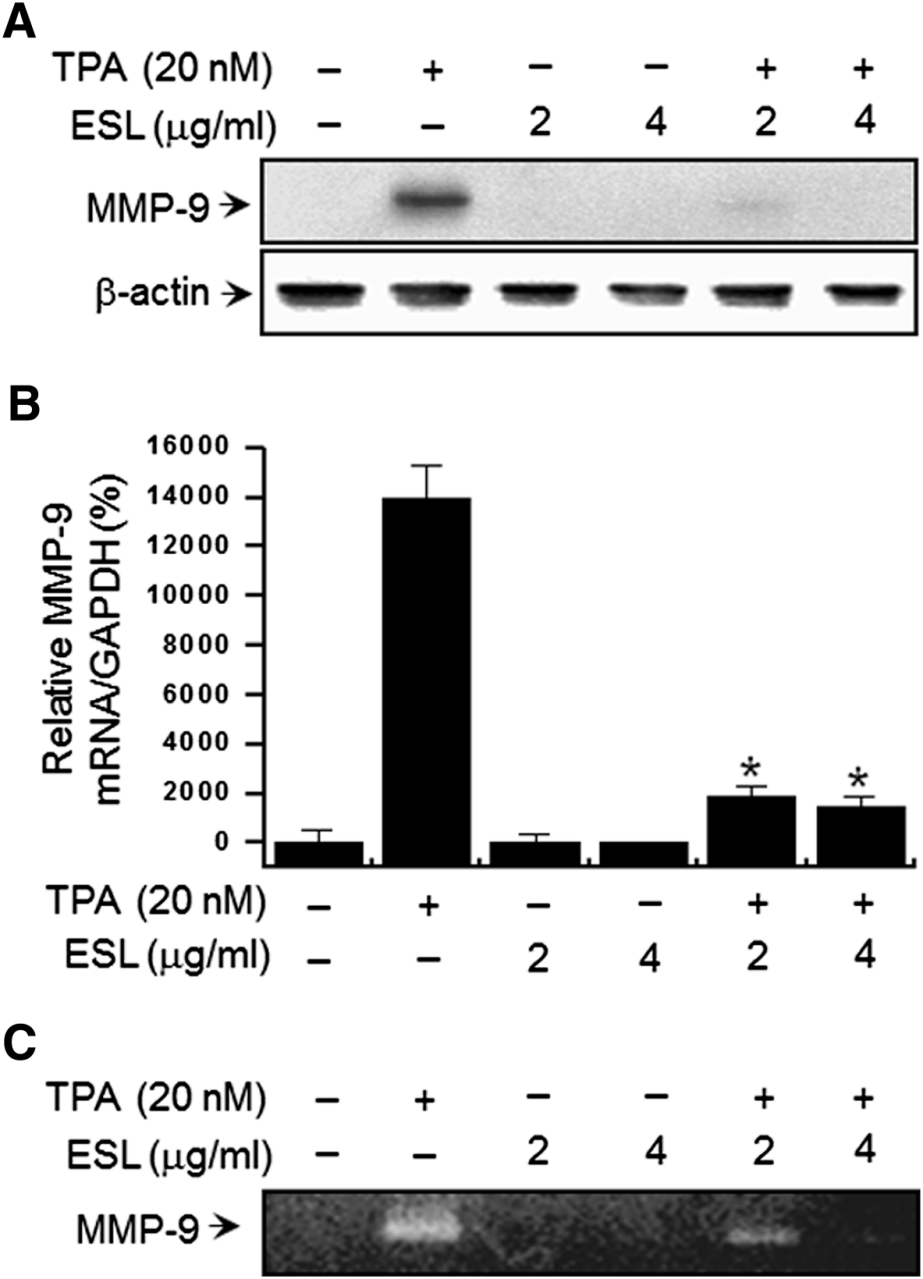
**The effect of ethanol extract of Saussurea lappa (ESL) on 12-O-tetradecanoylphorbol-13-acetate (TPA)-induced metalloproteinase-9 (MMP-9) expression in MCF-7 cells.** MCF-7 cell monolayers were treated with the indicated ESL concentrations in the presence of TPA for 24 h. **(A)** Cell lysates were analyzed by western blot with anti-MMP-9 antibody. The blot was reprobed with anti-β-actin to confirm equal loading. **(B)** MMP-9 mRNA levels were analyzed by real-time PCR and glyceraldehyde 3-phosphate dehydrogenase (GAPDH) was used as an internal control. **(C)** Conditioned medium was prepared and used for gelatin zymography. Each value represents the mean ± SE of three independent experiments. *p < 0.01 vs. TPA.

To investigate the effect of ESL on TPA-induced MMP-9 expression, we performed western blot analysis, RT-PCR, and zymography in MCF-7 cells. Western blot analysis revealed that ESL treatment of MCF-7 cells blocked the upregulation of MMP-9 protein expression by TPA (Figure 2A). RT-PCR revealed that TPA increased the MMP-9 level in MCF-7 cells, and that ESL significantly inhibited this upregulation (Figure 2B). To further investigate the effect of ESL on the TPA-induced MMP-9 secretion in MCF-7 cells we performed a zymography assay. MCF-7 cell treatment with TPA resulted in increased MMP-9 secretion, which was significantly diminished by ESL treatment (Figure 2C).

### Effect of ESL on the TPA-induced NF-κB and AP-1 DNA binding activity

**Figure 3:**
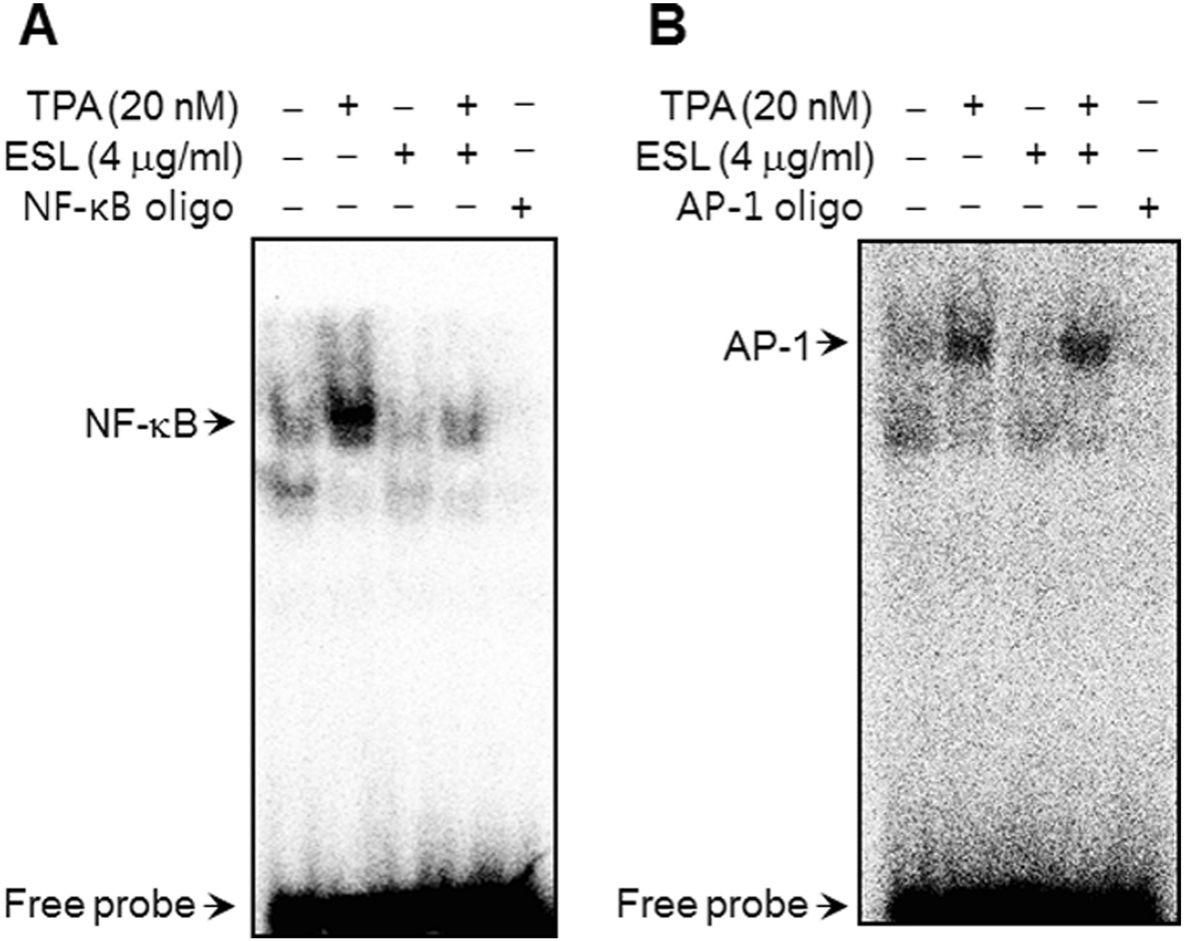
**The effect of ethanol extract of Saussurea lappa (ESL) on the 12-O-tetradecanoylphorbol-13-acetate (TPA)-induced nuclear factor-kappa B (NF-κB) activation in MCF-7 cells.** MCF-7 cells were treated with ESL in the presence of TPA. Following 3 h of incubation, nuclear extracts were prepared as described in Methods. **(A, B)** NF-κB/activator protein-1 (AP-1) DNA binding was analyzed by electrophoretic mobility shift assay as described in Methods.

**Figure 4:**
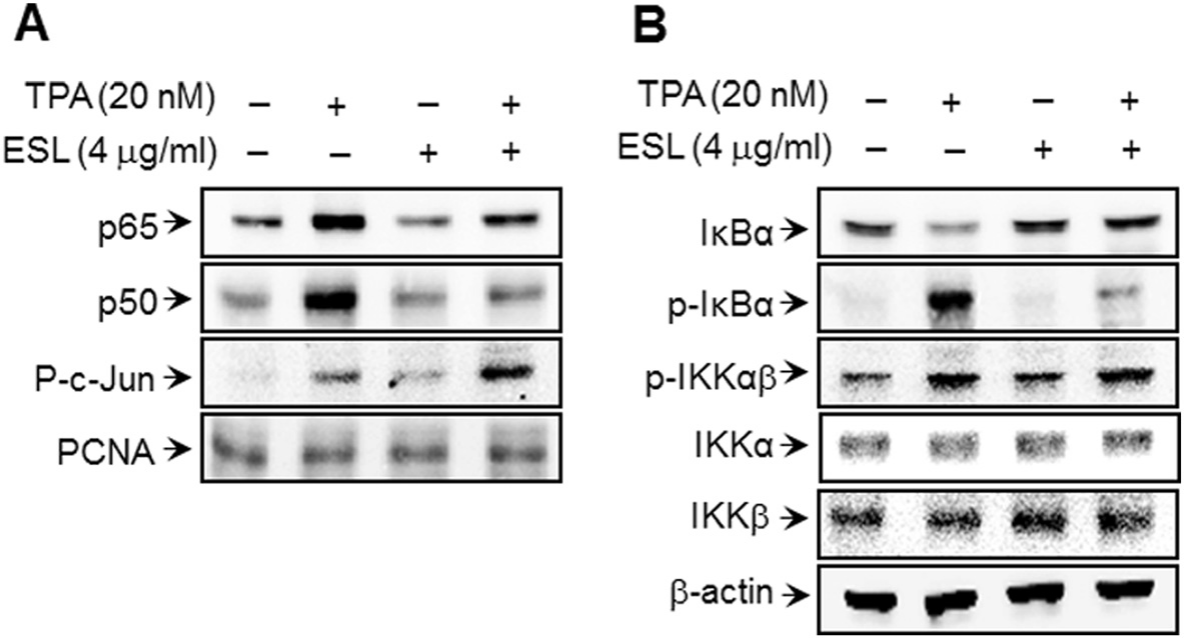
**The effect of ethanol extract of Saussurea lappa (ESL) on the 12-O-tetradecanoylphorbol-13-acetate (TPA)-induced nuclear factor-kappa B (NF-κB)-related signal proteins in MCF-7 cells.** MCF-7 cells were treated with ESL in the presence of TPA. Following 3 h of incubation, nuclear and cytoplasmic extracts were prepared. **(B)** Western blot analysis was used to determine the cytoplasmic levels of phosphorylated I-kappa B kinase-alpha/beta (p-IKKα/β), I-kappa B kinase-alpha (IKKα), I-kappa B kinase-beta (IKKβ), phosphorylated I-kappa B-alpha (p-IκBα), and I-kappa B-alpha (IκBα); and **(A)** the nuclear levels of NF-κB (p50 and p65 subunits) and the phosphorylated c-Jun subunit of activator protein-1 (AP-1).

To clarify the mechanism of the ESL-mediated inhibition of MMP-9 expression, the effect of ESL on the TPA-induced activation of NF-κB and AP-1 were evaluated using EMSA. As shown in Figure 3, TPA substantially increased the DNA binding activities of NF-κB and AP-1. Pre-treatment with ESL inhibited the TPA-stimulated NF-κB binding activity, but not the AP-1 binding activity, suggesting that ESL specifically blocks NF-κB activation in MCF-7 cells (Figure 3). Under normal biological conditions, the phosphorylation of IKKα/β induces the phosphorylation of IκBα. The cytoplasmic protein IκBα then binds directly to the p65 and p50 subunits of NF-κB and represses their nuclear translocation. We therefore proceeded to assess the changes in p-IKKα/β, IKKα, IKKβ, p-IκBα levels in the cytoplasmic fraction and p-c-Jun, p65, p50 levels in the nuclear fraction of ESL-treated and untreated MCF-7 cells. Our results demonstrate that the increased IκBα degradation and the translocation of p65 and p50 as a result of TPA stimulation are significantly suppressed by treatment with ESL, whereas p-IKKα/β, IKKα/β, and p-c-Jun levels were unaffected by TPA treatment (Figure 4). These findings indicate that the inhibition of TPA-induced MMP-9 expression by ESL occurs via NF-κB activation.

### MAPK signaling pathways are not involved in the inhibition of TPA-induced MMP-9 expression and secretion by ESL

**Figure 5:**
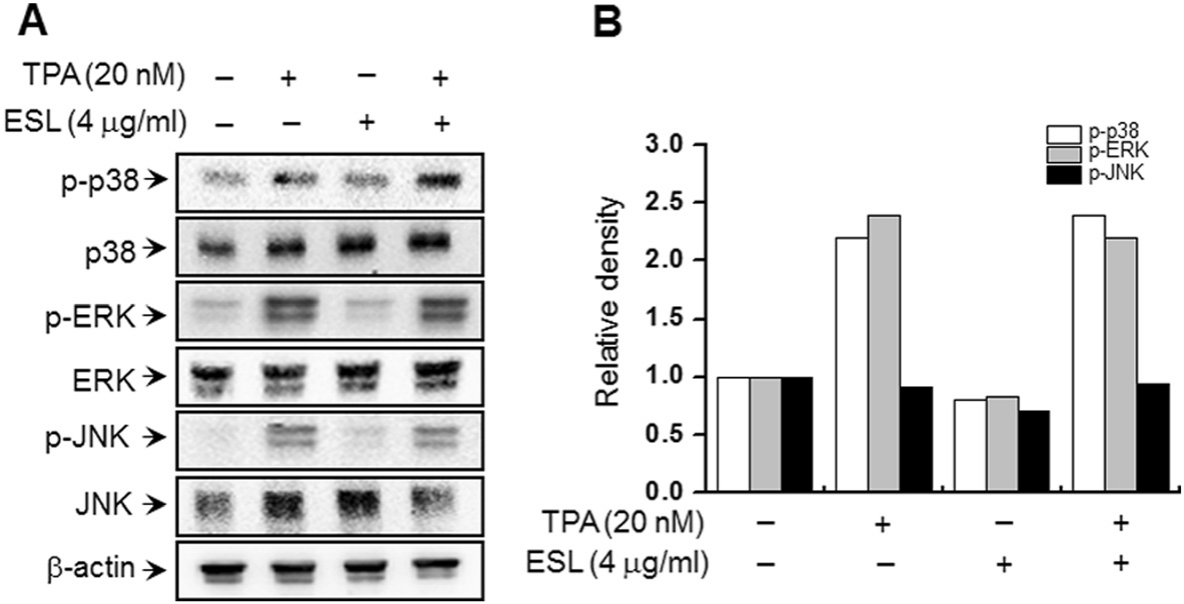
**The effects of ethanol extract of Saussurea lappa (ESL) on the 12-O-tetradecanoylphorbol-13-acetate (TPA)-induced mitogen-activated protein kinase (MAPK) signaling pathway in MCF-7 cells.** Cells were pretreated with TPA for 15 min in the presence or absence of ESL. **(A)** Cell lysates were prepared for western blotting with specific p-p38, p38, c-Jun N-terminal kinase (JNK), phosphorylated-JNK, extracellular signal-regulated kinase (ERK), and phosphorylated-ERK antibodies. **(B)** Quantitative results of phosphorylation levels of p38, ERK and JNK were adjusted with total p38, ERK and JNK protein level.

The activation of MMP-9 expression by MAP kinases (ERK, p38, and JNK) is widely recognized, and is known to occur via upstream modulation of NF-κB [17,29]. To investigate which particular MAPK is inhibited, the effect of ESL on the TPA-induced activation of MAPK was assessed using western blot analysis. As shown in Figure 5, the treatment of MCF-7 cells with TPA significantly enhanced the phosphorylation of p38, ERK, and JNK, and treatment of cells with ESL does not block this TPA-induced phosphorylation of the three kinases.

**Figure 6:**
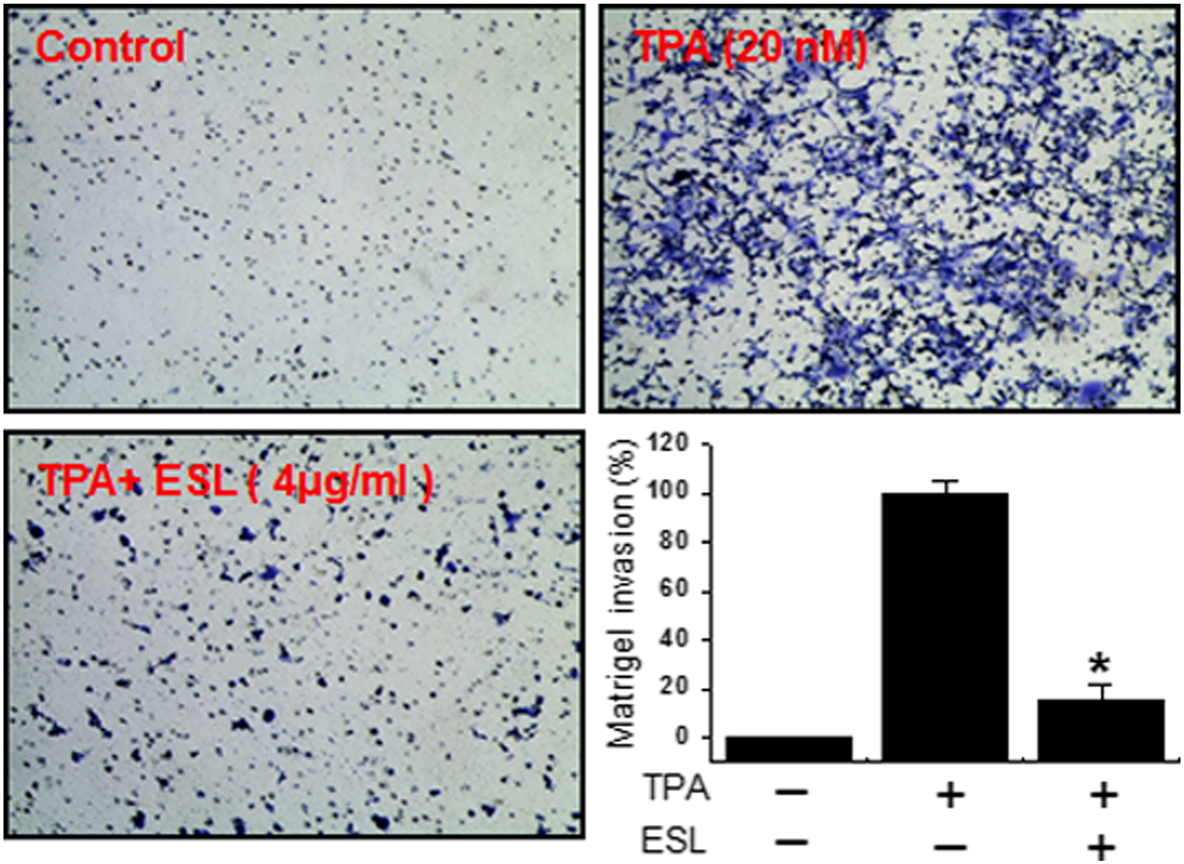
**The effects of ethanol extract of Saussurea lappa (ESL) on 12-O-tetradecanoylphorbol-13-acetate (TPA)-induced Matrigel**^**™ **^**invasion in MCF-7 cells.** MCF-7 cells were seeded into the upper chambers of Transwell plates and drugs placed in the wells. After 24 h incubation, cells on the bottom of the membrane were fixed, stained and counted. Each value represents the mean ± SE of three independent experiments. *p < 0.01 vs. TPA.

*Effect of ESL on the TPA-induced Invasion of MCF-7 Cells* In Vitro*.* It has been reported that the up-regulation of MMP-9 expression contributes to the invasiveness of cancer cells [2,30]. An in vitro invasion assay was carried out to investigate the effects of ESL on the invasive potential of MCF-7 breast adenocarcinoma cells. Treatment with TPA increased MCF-7 cell invasion when compared with untreated control cells, as determined by a Matrigel™ invasion assay. Incubation of MCF-7 cells with TPA resulted in a 10-fold increase in cell invasiveness, and treatment with ESL diminished this TPA-induced cell invasion by 85% (Figure 6).

## Discussion

The findings of this study provide the first evidence that ESL inhibits the TPA-induced expression of MMP-9 in MCF-7 breast cancer cells. Furthermore, we show the molecular mechanism of this inhibition to be a disruption of TPA-mediated activation of the NF-κB pathway, a finding in agreement with previous research, which has demonstrated that NF-κB is molecular target in ESL treated cells [28].

Breast cancer remains one of the most threatening mortality factors in women throughout the world despite the significant advancements in its early detection techniques and relevant therapeutic approaches. With a current mortality rate of 40%, over one million women worldwide will fall victim to breast cancer. Metastasis is the primary cause of breast cancer mortality. Tumor metastasis is a complex, multistep process that includes cell proliferation, ECM degradation, cell migration, and tumor growth at metastatic sites [13]. Morphologically, tumor invasion is associated with the presence of a distorted edge of the primary tumor, where individual or cohorts of tumor cells actively invade the tissue ECM surrounding the primary tumor [31]. MMP-9 is important for tumor metastasis because of its role in basement membrane cleavage, which allows migratory phenotype cells to be more invasive and motile [32-34]. In previous reports, inflammatory cytokines, growth factors, and phorbol esters were shown to stimulate MMP-9 by activating different intracellular signaling pathways in breast cancer cells [35-37]. The inhibition of MMP-9 expression might therefore be important in the development of a therapies for tumor metastasis.

To understand the TPA-induced signaling cascade underlying MMP-9 expression in MCF-7 cells, we assessed the effects of ESL on three MAPKs and the DNA binding abilities of transcription factors. The three major MAPK families, JNK, ERK, and p38 kinase, are expressed in MCF-7 cells, and their active phosphorylated forms can be detected [13]. MAPK signaling pathways are important for NF-κB and AP-1 activation, which require IκB kinase, PI3K-Akt, or p38 MAPK, depending on the cell type [11,19,38]. In addition, it has been established that TPA induces the nuclear transcription factors NF-κB and AP-1 in MCF-7 cells. NF-κB and AP-1 are important in regulating MMP-9, as the MMP-9 gene promoter contains NF-κB and AP-1 binding sites [17]. The NF-κB and AP-1 elements are centrally involved in TPA-mediated MMP-9 gene induction [15,29]. Our results, however, show that ESL inhibits the activation of NF-κB but not of MAPK or AP-1 in MCF-7 cells. This suggests that ESL inhibits TPA-induced MMP-9 expression via the NF-κB pathway.Our findings confirm that TPA-stimulated cell invasion is suppressed by ESL as demonstrated by the Matrigel™ invasion assay showing inhibition of the TPA-induced invasion potential of MCF-7 cells by ESL (Figure 6).

## Conclusions

Our results demonstrated that ESL is a potent inhibitor of TPA-induced MMP-9 expression and strongly blocks the action of the NF-κB signaling pathway in MCF-7 cells. This is the first report demonstrating the suppression of TPA-stimulated cancer cell invasion by inhibition of MMP-9 expression via suppression of NF-κB pathways in MCF-7 cells.

## Competing interests

The authors declare that they have no competing interests.

## Authors’ contributions

KBK, YRL conceived and designed the study. HRK, JMK JKH and YJR performed the experiments. MSK, YJP and SHY analyzed the data. KBK and YRL drafted the manuscript. HJK DGR, BSM and JMY provided comments and editorial review of the manuscript. DSL, HO and YCK provided the Saussurea lappa extracts. All authors read and approved the final manuscript.

## Pre-publication history

The pre-publication history for this paper can be accessed here:

http://www.biomedcentral.com/1472-6882/14/170/prepub
